# Highly efficient upconversion of Er^3+^ in Yb^3+^ codoped non-cytotoxic strontium lanthanum aluminate phosphor for low temperature sensors

**DOI:** 10.1038/s41598-017-17725-z

**Published:** 2017-12-15

**Authors:** K. Pavani, J. Suresh Kumar, K. Srikanth, M. J. Soares, E. Pereira, A. J. Neves, M. P. F. Graça

**Affiliations:** 1 Department of Physics & I3N, University of Aveiro, 3810-193 Aveiro, Portugal; 2 CESAM-Centre for Environmental and Marine Studies & Department of Chemistry, University of Aveiro, 3810-193 Aveiro, Portugal

## Abstract

Er^3+^ and Er^3+^/Yb^3+^ melilite-based SrLaAl_3_O_7_ (SLA) phosphors were synthesized by a facile Pechine method. The differences in emission intensities of ^4^I_13/2_ → ^4^I_15/2_ transition in NIR region when excited with Ar^+^ and 980 nm lasers were explained in terms of energy transfer mechanisms. Temperature and power dependence of upconversion bands in the visible region centered at 528, 548 and 660 nm pertaining to ^2^H_11/2_, ^4^S_3/2_ and ^4^F_9/2_ → ^4^I_15/2_ transitions were investigated. Fluorescence intensity ratio (FIR) technique was used to explore temperature sensing behaviour of the thermally coupled levels ^2^H_11/2_/^4^S_3/2_ of Er^3+^ ions in the phosphors within the temperature range 14–300 K and the results were extrapolated up to 600 K. Anomalous intensity trend observed in Er^3+^ doped SLA phosphor was discussed using energy level structure. Cytotoxicity of phosphors has been evaluated using 3-(4,5-dimethylthiazol-2-yl)-2,5-diphenyltetrazolium bromide (MTT) assay in Bluegill sunfish cells (BF-2). The non-cytotoxic nature and high sensitivity of the present phosphors pay a way for their use *in vitro* studies and provide potential interest as a thermo graphic phosphor at the contact of biological products.

## Introduction

Recent interest on rare earth (RE^3+^) photoluminescence (PL) is not only because of the efficient down conversion (DC) but also about the wide range application of their upconversion (UC) in the fields of color displays, LEDs, bio-imaging, temperature sensors etc.^1–5^. Fluorescence intensity ratio (FIR) technique is widely used for the temperature sensors based on glasses/glass-ceramics doped with RE^3+^, providing high detection spatial resolution, precision and excellent sensitivity^5–7^. As for concerned with low temperature sensors, CdSe, CdTe semiconductor quantum dots were used as optical temperature nanoprobes as their peak emission wavelength changes with the temperature^8^, but are of limited application due to low intensity along with difficult preparation procedures. RE^3+^ doped glasses and glass-ceramics also suffer from low intensity and poor performance. One of the important conditions a material should satisfy in order to be used as temperature sensor is that the radiative transitions from a fluorescent level should dominate its non-radiative transitions. This could be achieved only when the host has considerably low maximum phonon energy. This condition is also relevant to achieve UC, since a material which has low maximum phonon energy can be effectively used for RE^3+^ UC.

In order to use an UC phosphor for bio-imaging, it is mandatory to have low cytotoxicity with low phonon energy. Though fluoride based phosphor materials are found to have low maximum phonon energy, their reported dubious nature concerned to cytotoxicity make the usage of these materials for biological applications suspicious^9^. In some studies, upconversion nanomaterials such as NaYbF_4_, NaYF_4_, etc. were found to be cytotoxic on different cell lines^10–12^, while in others they are found to be non-cytotoxic^13–15^. Usually different coatings are mandatory to make the fluoride particles non-cytotoxic. In view of the uncertain toxic nature of different low maximum phonon energy based materials like fluorides, it would be even better to have a material with low toxicity by itself without a non-toxic coating^16,17^. Bio-imaging is normally done at low temperatures or at room temperatures and hence estimation of the temperature of a biological product at the surface would be essential. Bio-materials are sensitive and absorb most of the radiation in the region of ultra violet (UV) and visible (Vis) region^18^. Hence UC materials with near infrared (NIR) excitation would be the best suitable materials for the temperature sensing at the contact of biological product.

Many of the trivalent lanthanides such as Er^3+^, Tm^3+^, Ho^3+^, Nd^3+^, Dy^3+^ and Eu^3+^ have been used as PL centres of optical thermometry. But the Er^3+^ ion has proved itself to be an efficient UC centre among RE^3+^ ions and tremendous increase of its efficiency when combined with Yb^3+^ ions, acting as sensitizer, makes it the ultimate choice among UC ions. The UC PL intensity ratio between ^2^H_11/2_ and ^4^S_3/2_ levels has been proved to be very efficient as the bands are due to two photon excitation and the ratio is very sensitive to the temperature^7^.

Strontium lanthanum aluminate, SrLaAl_3_O_7_ (SLA) is a complex compound that crystallizes in the tetragonal melilite-like structure with a space group P42_1_m with a general chemical formula ABC_3_O_7_ (where A = Ca, Sr, Ba; B = La, Gd, Y and C = Al, Ga)^19,20^. The crystal structure of SLA is composed of five membered rings of AlO_4_ tetrahedra linked at each corner, with the Sr^2+^ and La^3+^ ions situated at the centres of these tetrahedra^20^. It is reported to have good mechanoluminescence properties and can be prepared with RE^3+^ ions as dopants in several methods^20–24^.

To use any of the phosphor particles in industrial production, the impacts of these particles are to be monitored scientifically for its safer development as technology-based product. The particles during their production, transport, use and disposal are seen entering into the environment^25,26^. The release of these particles into the aquatic environment as ‘an ultimate sink’ cannot be denied and can pose serious risk to the inhabiting organism^27^. Cytotoxicity evaluation is an important tool to understand the particles impact on the aquatic environment. The BF-2 cells derived from the caudal fin tissue of the bluegill sunfish (*Lepomis macrochirus*), is extensively used in both *in vivo and in vitro* toxicity evaluation^28,29^. Moreover, recent investigations show that the MTT (3-(4,5-dimethylthiazol-2-yl)-2,5-diphenyltetrazolium bromide) assay has been widely used and remains the most popular assays to be relied in academic and research works to know the material’s cytotoxic nature^30,31^.

As a variety of cellular events are effectively marked by temperature changes, a way to monitor the temperature of biological materials by a non-contact method is very essential^32^. This requirement is the base of our investigation in which non-cytotoxic oxide based material with low phonon energy has been investigated for its application in temperature sensing applications in biological materials. We describe the experiments done to evaluate the application of SLA doped with Er^3+^ ion or with the Er^3+^-Yb^3+^ ion pair, synthesized by Penchini process, to monitor the temperature by FIR method. The UC PL of SLA:Er and SLA:Er/Yb phosphor were studied at various laser pump powers and temperatures. The temperature dependent behavior of the PL intensity and the ratio of PL bands were studied, and the thermometry characteristics were evaluated. *In vitro* cytotoxicity of SLA phosphor particles has also been evaluated using BF-2 fish cell line and MTT assay^28^.

## Results and Discussion

### Structural and morphological studies

**Figure 1:**
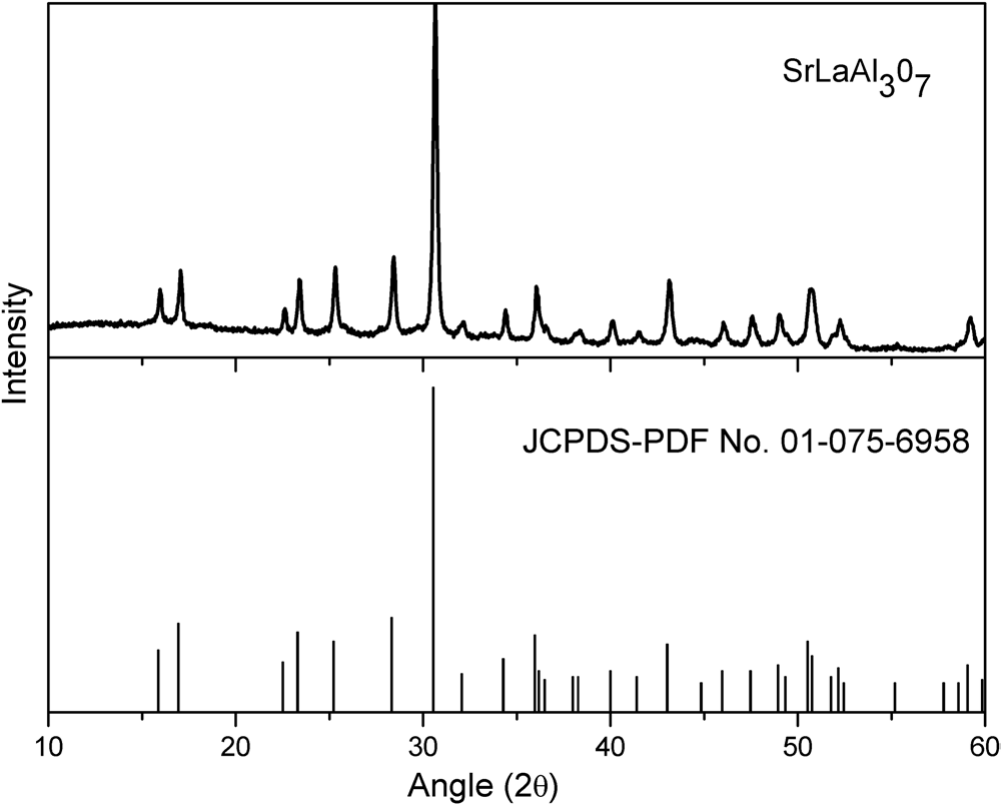
X-Ray Diffraction pattern of SrLaAl_3_O_7_ host material.

The phase purity and crystal structure of the host SrLaAl_3_O_7_ (SLA) was identified using XRD. The pattern contains pure tetragonal phase of SLA matrix with space group *P42*_1_ *m* as shown in Fig. 1. The position of all diffraction peaks match well compared to the standard data of SLA (JCPDS no. 01-075-6958). The crystallite size of the phosphors were calculated using the Debye-Scherrer equation^33^, *D* = *kλ* / (*B* cos *θ*), where *D* is the average grain size, *k* (0.9) is the shape factor of the average crystallite, *λ* represents the Kα_1_ radiation wavelength of copper, *B* is the full width at half maximum (FWHM) of the diffraction peak, and *θ* represents the angle of diffraction, i.e., half of the diffraction angle 2*θ*. The estimated crystallite size is found to be 65 nm and the unit cell parameters for tetragonal phase is calculated with Unit Cell software are *a* = b = 7.895 Å and *c* = 5.217 Å with *V* = 325.21 Å^3^.

**Figure 2:**
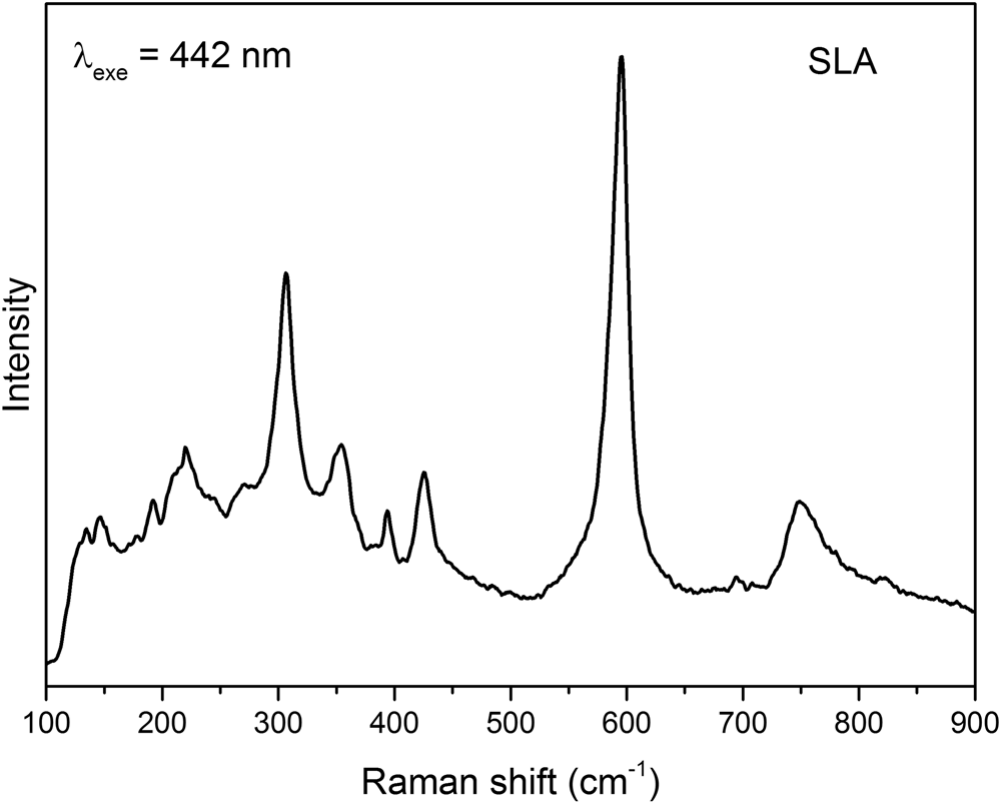
Raman scattering spectrum of SrLaAl_3_O_7_ material upon 442 nm excitation.

**Figure 3:**
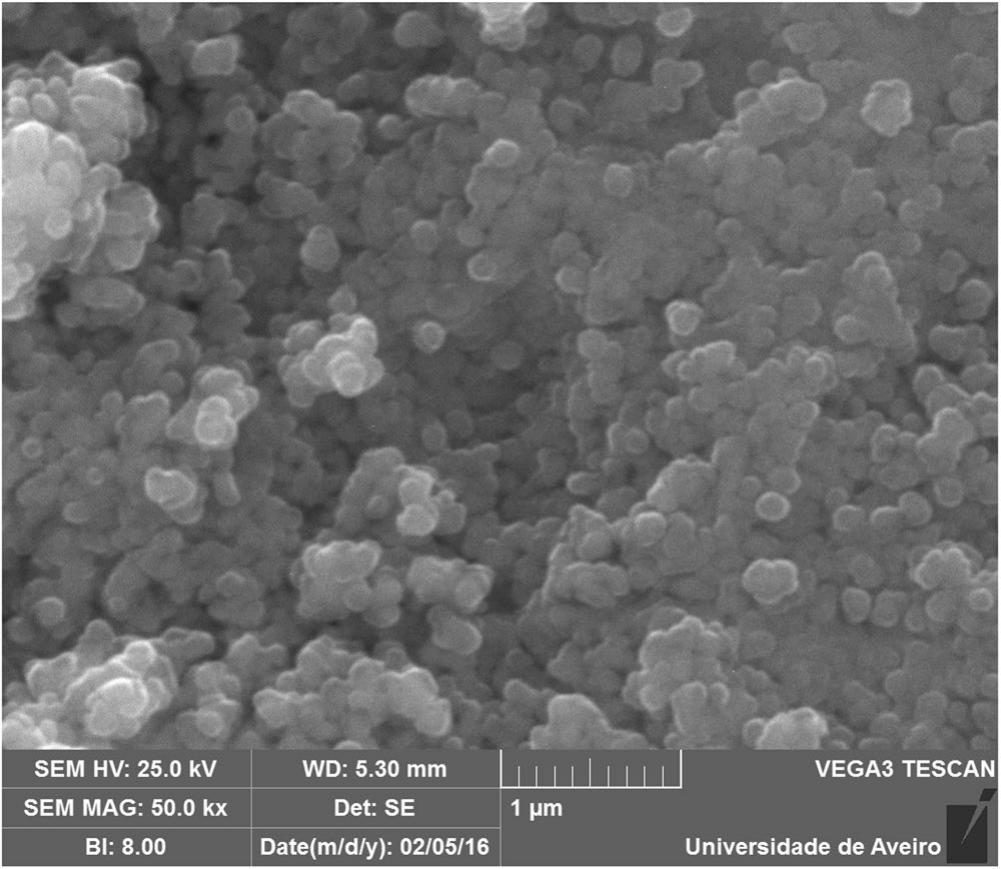
Scanning electron microscopic image of SrLaAl_3_O_7_ host powder.

Figure 2 shows the Raman spectrum of the prepared SLA host material that consists of totally ten major bands. The spectrum is dominated by a major intensity peak at 596 cm^−1^. Studies on Strontium lanthanum aluminate compounds suggest that the band falling in the 585–595 cm^−1^ region seems to be originated as a consequence of local distortion of AlO_6_ octahedron and / or other defects in the matrix^34–36^. In low wavenumber region below 400 cm^−1^, the spectrum possess several overlapped modes at 146, 219, 275 and 306 cm^−1^ that are attributed to vibrational modes of La/Sr–O and the bridge Sr/La - O - Sr/Al oxygen atoms, respectively^34–37^. The SEM image of SLA host is shown in Fig. 3. Average particle size were in the range of 200–400 nm. The particles are in spherical shape and tend to aggregate.

### NIR photoluminescence

**Figure 4:**
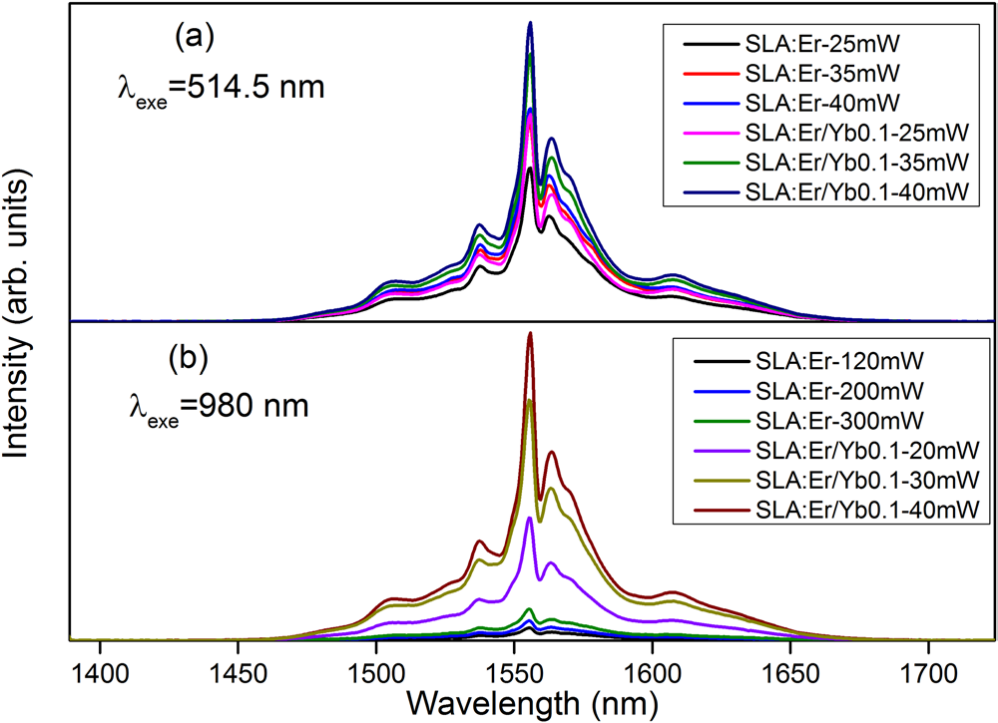
NIR emission spectra of SLA:Er/Yb0.1 phosphor upon (**a**) 514.5 nm and (**b**) 980 nm excitation at different powers.

Under 514.5 and 980 nm excitation the NIR emission spectra for SLA:Er and SLA:Er/Yb0.1 samples exhibits a broad emission band attributed to ^4^I_13/2_ → ^4^I_15/2_ transition of Er^3+^ ions as shown in Fig. 4 (a) and (b) respectively. The broad band spanning from 1450–1675 nm with a maxima at 1556 nm displays large number of splittings due to the crystal field effect of SLA matrix.

The Ar^+^ laser radiation (514.5 nm) excites Er^3+^ ion to ^2^H_11/2_ state, from where it readily de-excite radiatively or non-radiatively (NR) reaching ^4^I_13/2_ level. The final radiative transition to the ground state generates the broad NIR peak (Fig. 4(a)). It is observed that as the power of the Ar^+^ laser radiation is increased, the emission of both phosphors shows the same structure, both rising in emission intensity. This behavior is according to that what it was expected; the 514.5 nm laser light excites directly the Er^3+^ ions, which is the activator in both the phosphors. As the concentration of Er^3+^ ions is same in both the phosphors there is only a nominal change in the intensity of the NIR PL, that can be attributed to the position and the alignment of the sample in the spectrometer.

**Figure 5:**
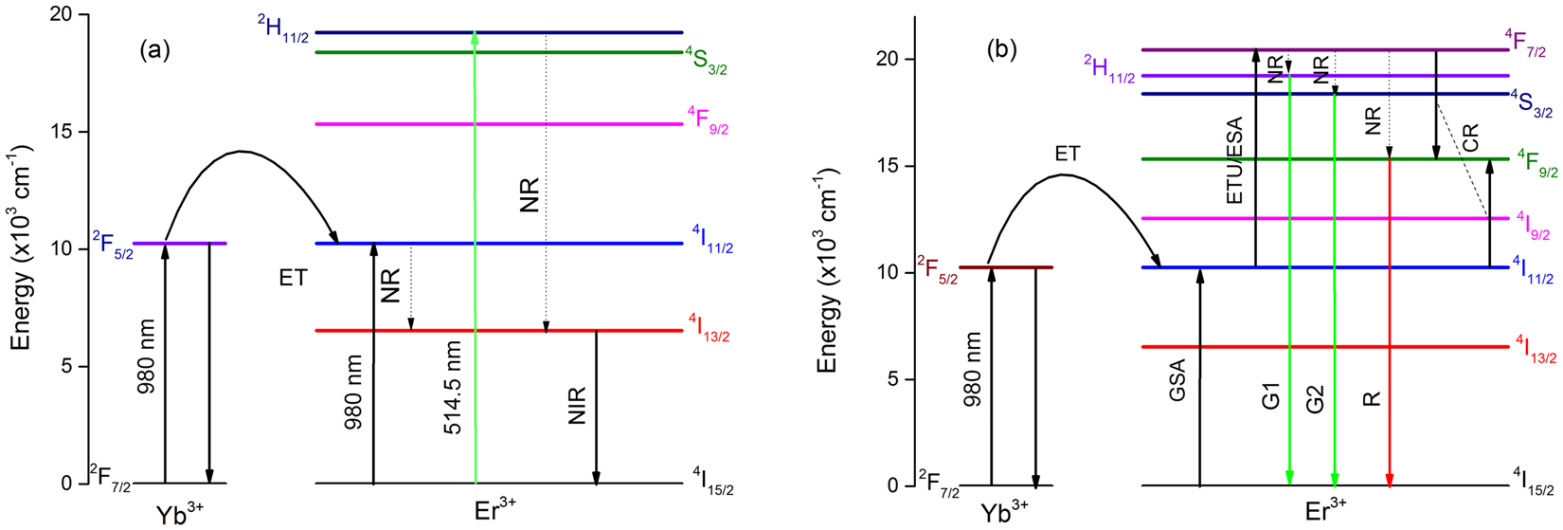
Partial energy level diagram showing different process involved during (**a**) NIR emission and (**b**) visible upconversion in SLA:Er/Yb phosphors.

Figure 4(b) shows the NIR PL of the phosphors when excited with the 980 nm laser light at different powers. Like the Ar^+^ laser excitation Fig. 4(a), the emission spectra of both powers exhibit a similar structure, but with a large difference in their intensities. Compared to the PL intensities of SLA:Er, PL intensities of SLA:Er/Yb0.1 phosphor are much higher although the laser powers are low in the later case. The 980 nm laser radiation excites both Er^3+^ and Yb^3+^ ions to ^4^I_11/2_ and ^2^F_5/2_ levels, respectively. As the absorption cross-section of Yb^3+^ ions is far high compared to Er^3+^ at this resonant wavelength^38^, more Yb^3+^ ions are excited, which intern transfers energy to Er^3+^ ions. Hence, more number of Er^3+^ are excited in SLA:Er/Yb0.1 compared to SLA:Er phosphor, thus radiating high intense NIR emission. The excitation mechanism, energy transfers, radiative and non-radiative process in SLA:Er and SLA:Er/Yb0.1 samples were shown in Fig. 5(a).

### Upconversion luminescence

**Figure 6:**
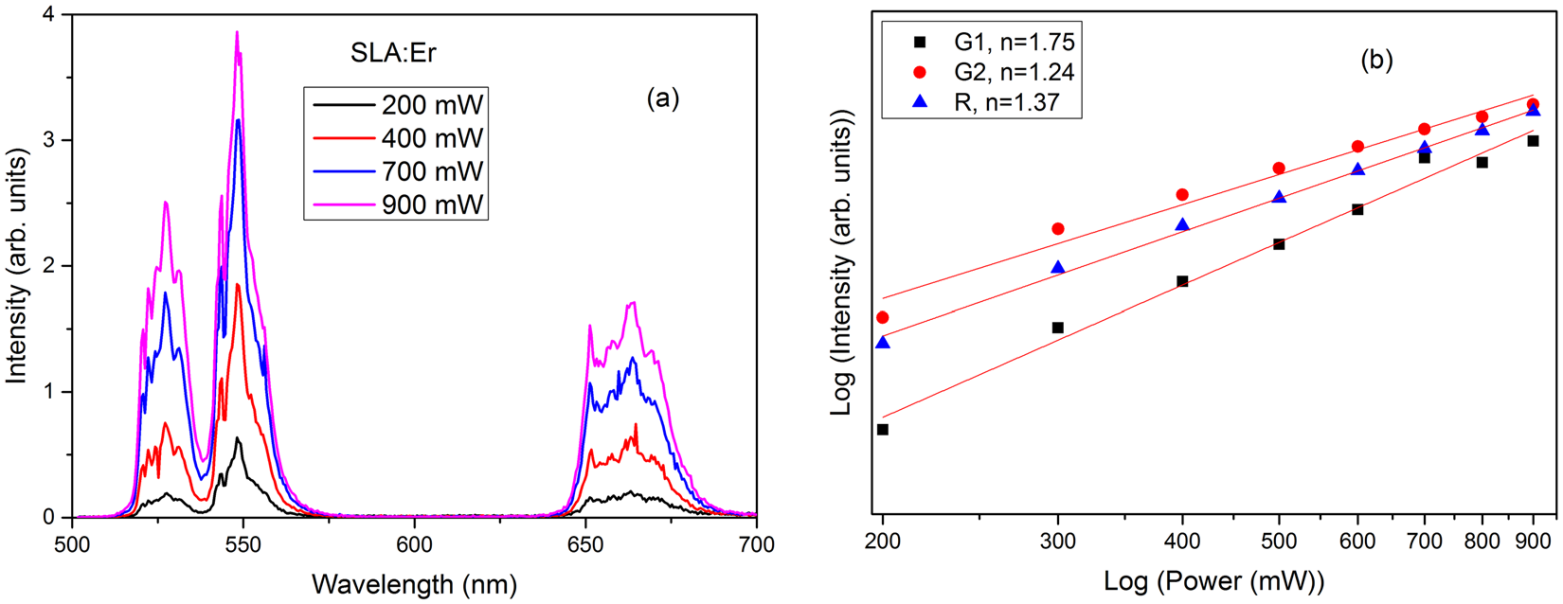
**(a**) Power dependent upconversion in SLA:Er phosphor upon 980 nm excitation and (**b**) Power vs upconversion intensity in logarithmic scale.

Figure 6(a) presents the power dependent UC emission spectra for SLA:Er phosphor. Totally there are three main UC bands in the visible region centered at 528 (^2^H_11/2_ → ^4^I_15/2_), 548 (^4^S_3/2_ → ^4^I_15/2_) and 660 nm (^4^F_9/2_ → ^4^I_15/2_), which are labeled by G1, G2 and R respectively and are schematically represented in Fig. 5(b). It is well known that UC process can occur through several mechanisms such as excited state absorption (ESA), ground state absorption (GSA) and energy transfer (ET). Initially Er^3+^ ions in SLA phosphor would be excited to ^4^I_11/2_ level through GSA, since the level possess longer lifetime, the same Er^3+^ ion excites to ^4^F_7/2_ level through ESA. The green and red emissions are the outcome of this ESA followed by phonon decay to the ^2^H_11/2_ or ^4^S_3/2_ level for G1 or G2 and further to ^4^F_9/2_ level for R emission. At all powers the G2 emission transition (^4^S_3/2_ → ^4^I_15/2_) is stronger than that of G1 emission transition (^2^H_11/2_ → ^4^I_15/2_). As shown in Fig. 6(a) each emission transition contain more than one component due to Stark splitting of Er^3+^:^2^H_11/2_, ^4^S_3/2_ and ^4^F_9/2_ levels^39^. For all laser powers the emission spectra of SLA:Er exhibited same features except increment in their intensities.

The UC emission and pump power dependence emissions are sensitive to dopant concentration. As the concentration of dopant (Er^3+^) ions is low, the trend of increment follows linear. At high concentration and high powers UC emission follows saturation phenomena due to depletion of pump power. It is well known that the intensity ‘*I’* of UC emission is proportional to some power ‘*n’* of excitation power ‘*p’*. The number *n* indicates the number of IR photons required to excite the luminescent ions to get the UC emission. The slope of line plotted with integrated emission intensity of the UC PL as a function of the pump power in logarithmic scale, gives the number of photons required for UC process. In Fig. 6(b), fitting of the data yields a straight line with a slope of approximately 1.75, 1.24 and 1.37 for ^2^H_11/2_ → ^4^I_15/2_, ^4^S_3/2_ → ^4^I_15/2_ and ^4^F_9/2_ → ^4^I_15/2_ transitions respectively, which proves that two photon process predominant in present SLA:Er phosphor

**Figure 7:**
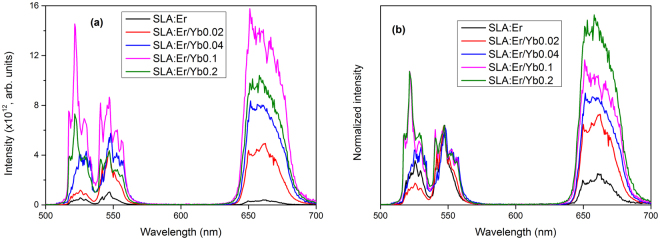
(**a**) Yb^3+^ concentration dependent upconversion in SLA:Er/Yb phosphors and (**b**) upconversion spectra normalized with respect to G2 band.

UC emission in singly Er^3+^ doped materials differs from that in Yb^3+^ sensitized samples because of the differences in energy transfer. Yb^3+^ ions possess large absorption cross-section for 980 nm photons, and as a result, energy transfer upconverson (ETU) process dominates in Er^3+^/Yb^3+^ codoped sample compared to ESA in singly Er^3+^ doped sample. The population accumulation in ^2^H_11/2_ and ^4^S_3/2_ states occur by successive ET process from the excited ^2^F_5/2_ state of Yb^3+^ ions to the Er^3+^ ions exciting them first to the ^4^I_11/2_ state and in a second step to the ^4^F_7/2_ excited state. Followed by a non-radiative (NR) relaxation process as shown in Fig. 5 (b), the Er^3+^ ions de-excite to ^2^H_11/2_ and due to a small energy gap between ^2^H_11/2_ and ^4^S_3/2_ states, the Er^3+^ ions can relax fast to the ^4^S_3/2_ state resulting in the observed two green emission bands G1 and G2. Alternatively, the ions can relax non-radiatively to the ^4^F_9/2_ level leading to red emission of R band. Figure 7(a) shows the UC emission spectra of SLA:Er and SLA:Er/Yb samples when excited with 980 nm light at similar experimental conditions to compare the intensities of spectra. It is observed from the figure that the intensity of UC of SLA:Er is very low compared to SLA:Er/Yb samples. The intensity of UC bands increase with increase of Yb^3+^ concentration until 0.1 mol after which the intensities decrease with increase of Yb^3+^ concentration. This may be due to probable energy migration among Yb^´3+^ ions due to higher/saturated concentrations. To compare the changes in relative intensities of all the three bands with increase of Yb^3+^ concentration, the spectra were normalized with respect to G2 band and are presented in Fig. 7(b). From Fig. 7(b), it can be noticed that with addition of Yb^3+^ ions in to SLA:Er sample, the intensity of G1 band decreased initially and then increased till 0.1 mol of Yb^3+^ ions. For the 0.2 mol of Yb^3+^ ions G1 band intensity stays same as of 0.1 mol with respect to G2 band. It is also noticed that the band widths of all the UC emission bands increased with the addition as well as increase of Yb^3+^ ions’ concentration. The case of R band intensity is different than those of G1 and G2. The increase of R band with Yb^3+^ concentration is more pronounced. This may be due to the cross-relaxation (CR) process ^4^F_7/2_ + ^4^I_11/2_ → ^4^F_9/2_ + ^4^F_9/2_ between nearby Er^3+^ ions due to availability of high number of excited Er^3+^ ions with effective energy transfer from Yb^3+^ in their own vicinities as shown in Fig. 5 (b) ^40,41^. The CR channel promotes accumulation of more number of Er^3+^ ions at ^4^F_9/2_ adding more intensity to R emission compared to G1 and G2. Further increase of Yb^3+^ concentration, beyond 0.2 mol may eventually quench the overall UC emission from SLA sample with the CR channel and energy migration phenomenon. From all the different UC of SLA:Er and SLA:Er/Yb samples, it is found that SLA:Er/Yb0.1 has intense emission property among all the prepared phosphors.

**Figure 8:**
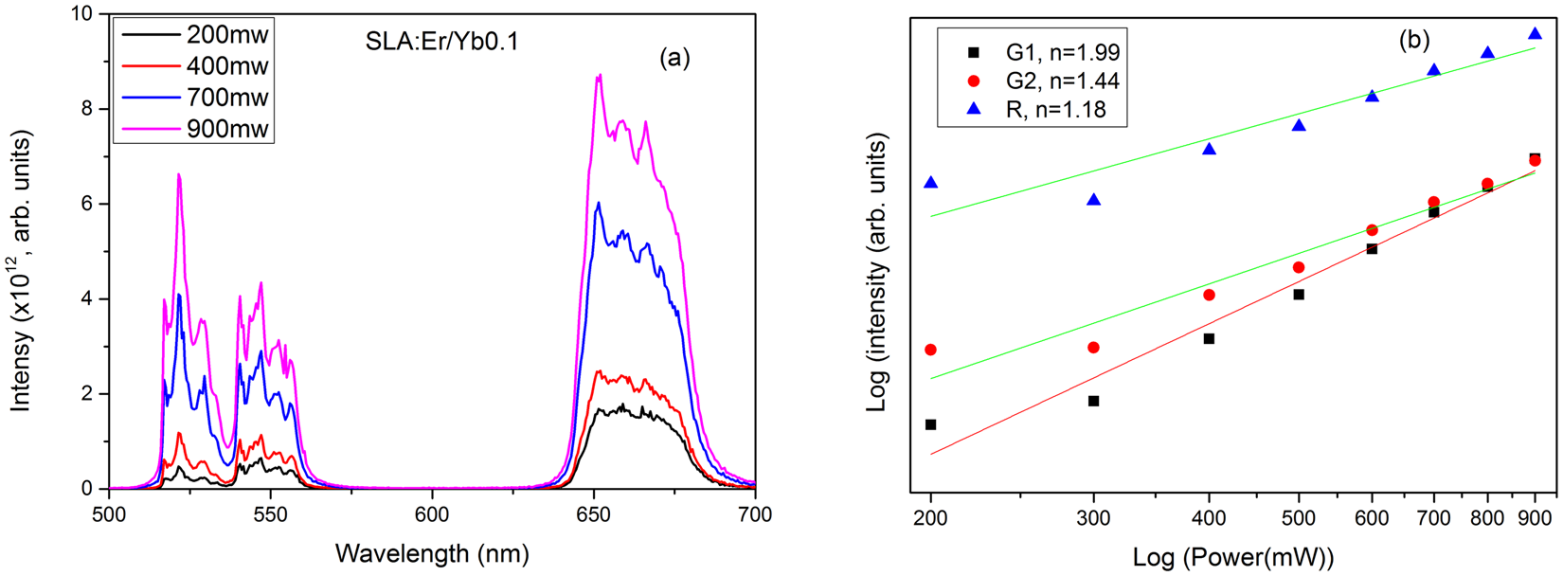
(**a**) Power dependent upconversion in SLA:Er/Yb0.1 phosphor upon 980 nm excitation and (**b**) Power vs upconversion intensity in logarithmic scale.

UC has also been recorded for SLA:Er/Yb0.1 sample by varying 980 nm laser power and is presented in Fig. 8(a). The intensity of the UC peaks increased with increase of laser power from 200–900 mW. The relative intensities of the UC bands in SLA:Er/Yb0.1 sample are not similar to SLA:Er sample because the red emission is dominant in SLA:Er/Yb UC. Figure 8(b) shows the logarithmic plot between UC band intensities and laser pump power. From the log-log plots, the slopes of the G1, G2 and R band intensities with power were found to be 1.99, 1.44 and 1.18, respectively. This signifies that there is involvement of two photons in the UC process and the low value of R band is due to the CR process which also prompts it to have a higher intensity compared to G1 and G2 bands.

### Temperature dependent upconversion emission and temperature sensing behaviour

**Figure 9:**
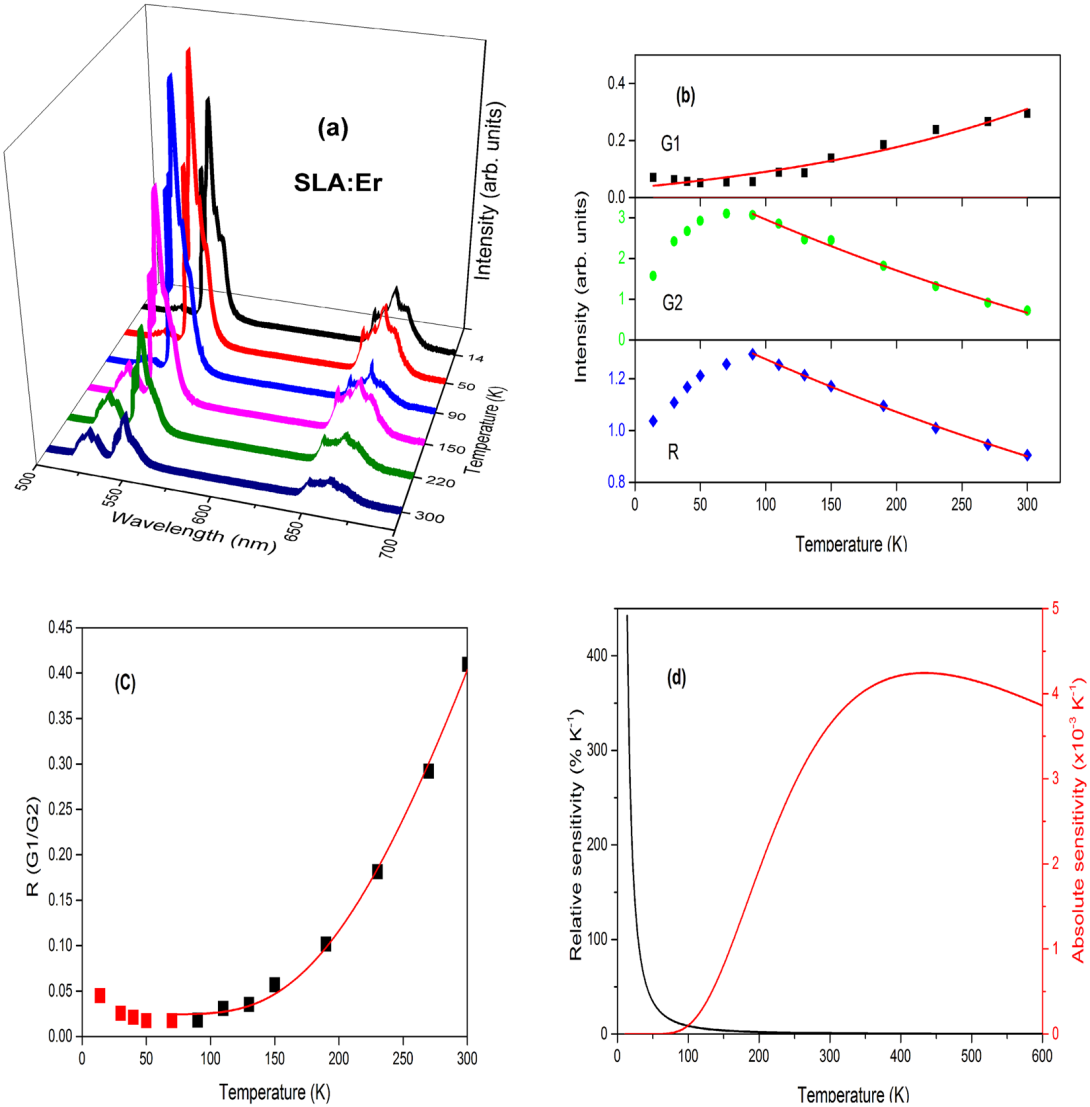
(**a**) Temperature dependent upconversion, (**b**) intensity trends, (**c**) FIR between G1 and G2 and (**d**) relative and absolute sensitivities of SLA:Er phosphor.

Temperature dependent UC emission spectra of SLA:Er phosphor is shown in Fig. 9(a). The intensities of G2 and R transitions show different behaviour, while G1 increases with increase of temperature. At low temperatures the intensity of G1 transition is almost negligible and increases exponentially with temperature, which is in good agreement to Boltzmann’s distribution law. According to Boltzmann’s distribution law the population at ^2^H_11/2_ level raises exponentially with increase of temperature compared to ^4^S_3/2_ level as the energy difference between these two levels is very small based on the formula

1 $$I(G1)/I(G2)={n}_{A}/{n}_{B}=C{\exp }(-{\rm{\Delta }}E/KT),$$

where *n*_*A*_, *n*_*B*_ are populations of considered energy levels, *C* is a constant which depends on the radiative transition rates and the degeneration of these levels and Δ*E* is the energy gap between the two energy levels. However, G2 and R transitions show a different behaviour. Their intensities first increase up to 90 K and then decrease with increase of temperatures. In comparison, the decrease of G2 is larger than R transition as shown in Fig. 9(b). The decrease in intensity above 90 K could be explained based on raise of multiphonon de-excitations of ^4^S_3/2_ and ^4^F_9/2_ levels which is quite common and known as thermal quenching. But the increase of G2 and R UC band intensities from lowest temperature to 90 K is due to the depopulation of higher sublevels of the ^4^I_11/2_ manifold which have a resonant match with the ^4^F_7/2_ level. Similar results were observed by I.R. Martin *et al*. and Van Der Ziel *et al*.^42,43^. According to Van Der Ziel *et al*., at low temperatures, after initial excitation of Er^3+^ ion from ^4^I_15/2_ to ^4^I_11/2_ level with 980 nm radiation, the excited ions occupy the lowest crystal field level of ^4^I_11/2_. Further, there would be a large mismatch of the energy to excite the ion at lowest crystal field level of ^4^I_11/2_ to ^4^F_7/2_ level. This mismatch leads to low intensity of UC bands at low temperatures. As the temperature increases, due to thermal agitation, occupancy of other crystal field levels pertaining to ^4^I_11/2_ increases that in fact decreases the mismatch to resonant transfer, leading to the increase in UC emission. However, the non-radiative rate increases rapidly at high temperatures and cause the radiative probability to decrease significantly. Therefore, the emission intensity decreases as temperature is increased beyond 90 K. Moreover, it is also observed that the intensity beyond 90 K decreased exponentially and hence the effect of energy mismatch was negligible as shown in Fig. 9(b).

FIR is one of the best techniques to determine the temperature sensitivity of a material to be used as thermographic phosphor. For this technique to be used for sensitivity, any active ion should have two adjacent energy levels which are thermally coupled. Any two adjacent energy levels will be called as thermally coupled levels (TCL) if they follow certain criteria. One of the important criteria which affect the efficiency of the ion for its sensitivity is the separation between these two TCL to be between 200 and 2000 cm^−1^. The other is the radiative transition of upper level should dominate over its non-radiative component^6^. There are two TCL in Er^3+^ ions, which can be used for FIR technique. One set is ^2^H_11/2_/^4^S_3/2_ and the other is ^4^D_7/2_/^4^G_9/2_. As the second set of Er^3+^ falls in UV region, its determination using UC is hindered with low signals. Hence the first set of TCL is the appropriate set to be used for FIR technique. FIR is defined as the ratio of the PL from each thermally coupled levels of active ions and can be expressed as^44,45^

2 $$FIR=Aexp[\frac{-{\rm{\Delta }}{E}_{f}}{KT}]+B$$

where *A* is a fitting constant, *B* is the overlapping of fluorescence peaks, Δ*E*_*f*_ is the fitting energy difference between the two thermally couples levels, *K* is Boltzmann’s constant and *T* is absolute temperature.

The absolute sensitivity of optical thermometry is the rate of change in the *FIR* in response to the temperature variation. Hence the absolute sensitivity (*S*_*A*_) of the TCL can be expressed as^44,46,47^.

3 $${S}_{A}=\frac{d}{dT}(FIR)=\frac{A{\rm{\Delta }}{E}_{f}}{K{T}^{2}}exp[\frac{-{\rm{\Delta }}{E}_{f}}{KT}]$$

To allow the comparison of different sensitivities of the materials, relative sensitivity has been derived by the following relation

4 $${S}_{R}\cong \frac{1}{FIR}\frac{d}{dT}(FIR)=\frac{{\rm{\Delta }}{E}_{f}}{K{T}^{2}}$$

Δ*E*_*f*_ is the fitting energy difference between the thermally coupled levels and the error with respect to experimental energy difference (Δ*E*_*m*_) gives an in site of its agreement with the experimental values. The error *δ*, can be expressed as^6,44,48,49^.

5 $$\delta =\frac{|{\rm{\Delta }}{E}_{f}-{\rm{\Delta }}{E}_{m}|}{{\rm{\Delta }}{E}_{m}}$$

**Figure 10:**
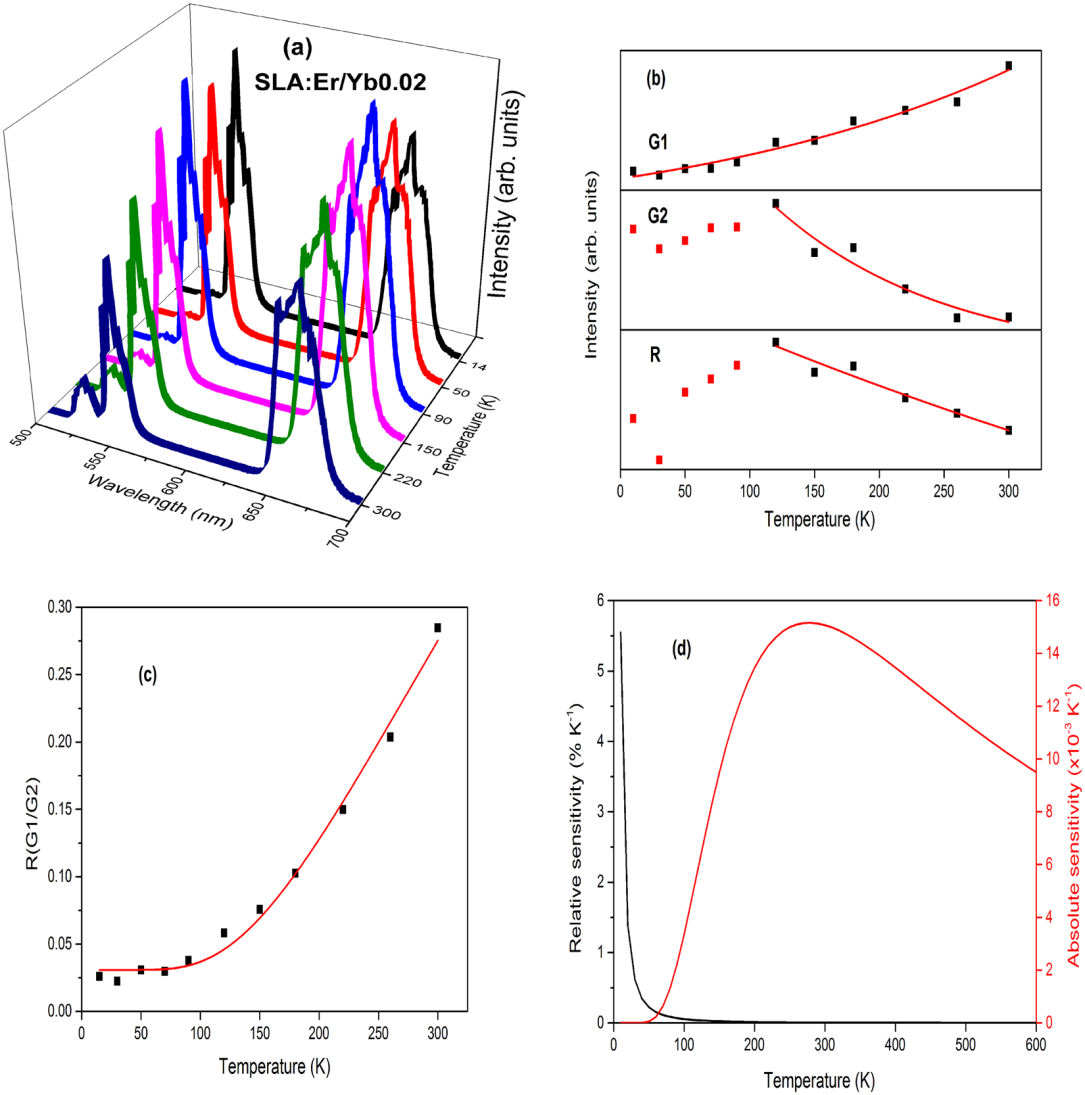
(**a**) Temperature dependent upconversion, (**b**) intensity trends, (**c**) FIR between G1 and G2 and (**d**) relative and absolute sensitivities of SLA:Er/Yb0.02 phosphor.

**Figure 11:**
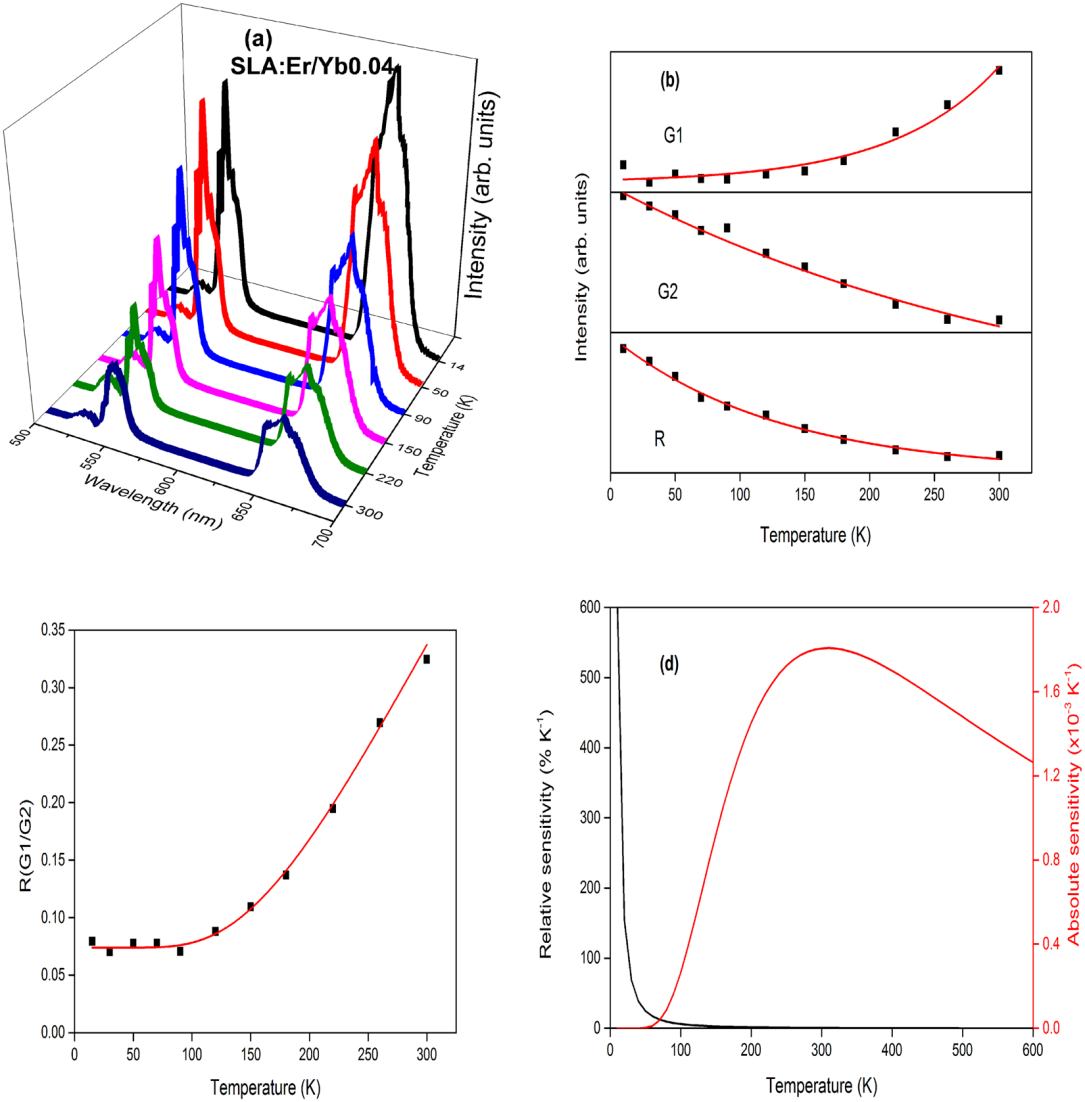
(**a**) Temperature dependent upconversion, (**b**) intensity trends, (**c**) FIR between G1 and G2 and (**d**) relative and absolute sensitivities of SLA:Er/Yb0.04 phosphor.

**Figure 12:**
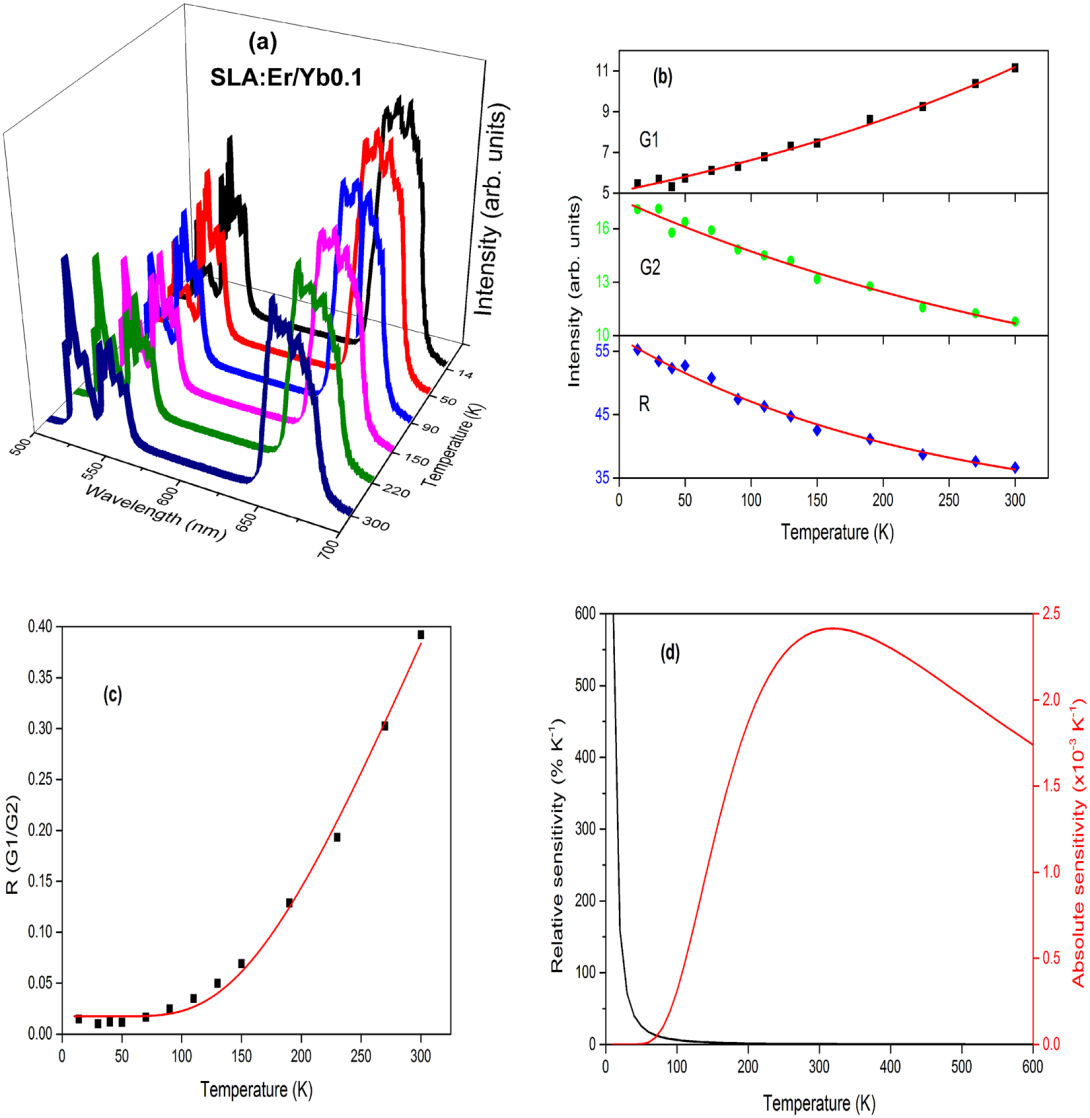
(**a**) Temperature dependent upconversion, (**b**) intensity trends, (**c**) FIR between G1 and G2 and (**d**) relative and absolute sensitivities of SLA:Er/Yb0.1 phosphor.

**Figure 13:**
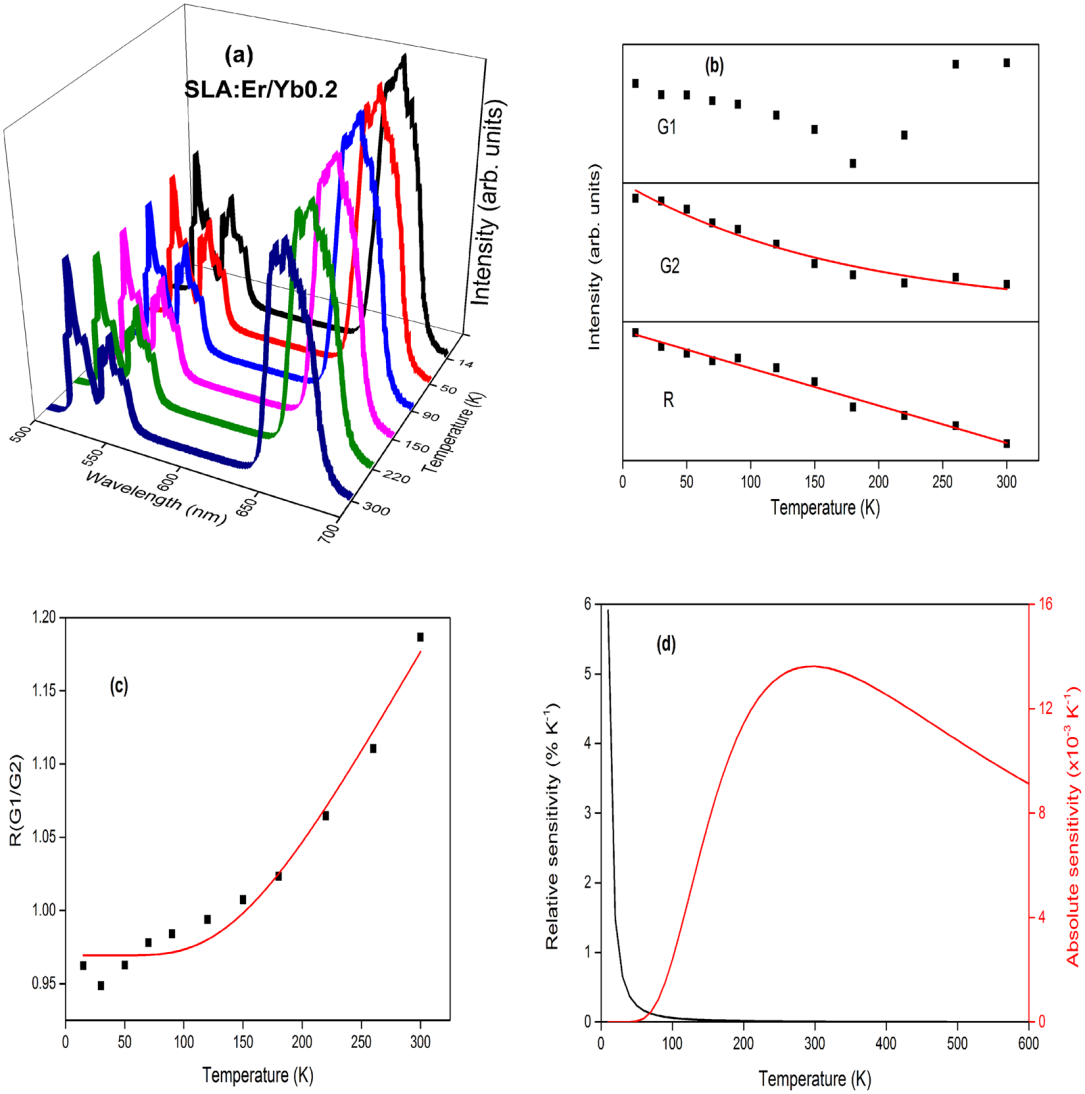
(**a**) Temperature dependent upconversion, (**b**) intensity trends, (**c**) FIR between G1 and G2 and (**d**) relative and absolute sensitivities of SLA:Er/Yb0.2 phosphor.

Figure 9(c) and (d) depict the FIR and relative as well as absolute sensitivities of SLA:Er phosphor. The FIR of the two peaks G1 and G2 had been fit using Eq. 2 and is shown in Fig. 9(c). Similarly, Figs 10–13 present temperature dependent UC (a), intensity trends (b), FIRs (c) and relative as well as absolute sensitivities (d) of SLA:ErYb0.02, SLA:ErYb0.04, SLA:ErYb0.1 and SLA:ErYb0.2 phosphors, respectively.

Except in case of SLA:Er/Yb0.02 sample, the UC emission spectra of all SLA:Er/Yb phosphors with respect to temperature show similar trend. As the temperature increases, the intensity of G1 bands increase exponentially, whereas with the increase of temperature, intensities of G2 and R decrease exponentially contrary to the trend observed for SLA:Er phosphor. This is explained based on the increase of phonon vibrations with the increase of temperature at high concentrations, eventually leading to decrease in UC intensities of G2 and R. In case of SLA:Er/Yb0.02 phosphor, the intensities of G2 and R (initial increase and decrease thereafter, with increase of temperature) show similar trend as of SLA:Er sample and this phenomenon may have occured because of low concentration of Yb^3+^ ions which could not compensate the large energy mismatch during effective UC process. For peak intensities of SLA:Er and SLA:Er/Yb0.02 samples, the fitting has been performed from 90 K as the typical intensity trends follow beyond that temperature. Whereas, for peak intensities of the remaining SLA:Er/Yb samples, the fitting has been performed from the lowest temperatures. In all the Figs 9–13(c), the black dots represent the ratio of intensities and the solid (red) line represents the fitting data. The parameter Δ*E*_*f*_, the fitting energy difference of the two peaks G1 and G2 for all the samples are presented in Table 1. Using Δ*E*_*f*_ and the fitting parameters of Eq. 2, absolute sensitivity (*S*_*A*_) and relative sensitivity (*S*_*R*_) have been calculated according to Eqs 3 and 4 for the UC data by extending the temperatures up to 600 K to correlate between all the phosphors and are also shown in Figs 9–13(d). Table 1 also presents complete fitting parameters including A, B and Δ*E*_*f*_ of Eq. 2, temperatures at which each sample exhibits highest *S*_*A*_, *S*_*R*_ at room temperature (297 K) and error between Δ*E*_*f*_ and Δ*E*_*m*_

**Table 1:** Exponential fitting parameters (A, B and ΔE_f_ **)** of fluorescence intensity ratios with respect to temperature, temperature of sample at highest absolute sensitivity (S_A_), relative sensitivity at 297 K and error (δ) between fitting as well as experimental energy difference pertaining to SLA:Er and SLA:Er/Yb samples.

Sample	A	B	ΔE_f_ (cm^−1^)	Temperature at highest S_A_ (K)	S_R_ at 297 K (% K^−1^)	δ
SLA:Er	6.43	0.02	590.41	440	0.98	0.15
SLA:Er/Yb0.02	1.55	0.03	385.60	280	0.65	0.44
SLA:Er/Yb0.04	2.06	0.07	429.14	310	0.71	0.38
SLA:Er/Yb0.1	3.06	0.01	443.00	320	0.75	0.36
SLA:Er/Yb0.2	1.48	0.96	411.01	300	0.65	0.40

It has been found that *S*_*A*_ is maximum at 440, 280, 310, 320 and 300 K for SLA:Er, SLA:Er/Yb0.02, SLA:Er/Yb0.04, SLA:Er/Yb0.1 and SLA:Er/Yb0.2, respectively. Maximum *S*_*A*_value temperature determines the temperature at which a material works with sensitively. The temperatures at which *S*_*A*_ values are maximum for the present UC phosphors show that they can be used at room temperature as well as at low temperatures effectively. The reliability of the temperature sensing can be made by the error, *δ* According to Eq. 5, the value of *δ* is found to be 0.15, 0.44, 0.38, 0.36 and 0.40 for SLA:Er, SLA:Er/Yb0.02, SLA:Er/Yb0.04, SLA:Er/Yb0.1 and SLA:Er/Yb0.2, respectively. Larger the value of *δ* for SLA:Er/Yb mean that the energy transfer between thermally coupled levels and other levels is not neglected and the population of thermally coupled levels at high temperature is induced by the routes of Boltzmann distribution which is essential for temperature sensing^6^. Hence, relatively larger values of *δ* values for SLA:Er/Yb phosphors over SLA:Er phosphor shows that the temperature sensing behaviour of SLA:Er/Yb phosphors can be relied more. In case of relative sensitivity, the *S*_*R*_ value is greater in SLA:Er samples than that of SLA:Er/Yb counterparts due to the greater value of Δ*E*_*f*_ for SLA:Er as shown in Table 1. Among SLA:Er/Yb phosphors with different Yb^3+^ concentrations, *S*_*R*_ increased with Yb^3+^ concentration until 0.1 mol and then decreased with further increase in Yb^3+^ concentration. SLA:Er/Yb0.1 has greater *S*_*R*_ value, higher temperature for maximum *S*_*A*_ value. Same phenomenon has been explored in relative intensities of UC with 980 nm at same excitation power as shown in Fig. 7(a). This means that the sample with highest UC intensity among different concentrations of Yb^3+^ ions would yield maximum *S*_*R*_ value at different temperatures. Moreover, the UC intensity of SLA:Er is too low to be used practically in comparision to the SLA:Er/Yb samples. From literature it is also evident that the sensitivity of SLA:Er and SLA:Er/Yb phosphors are also in accordance to the low phonon hosts such as fluorides, rare earth oxides etc that are highly used in bio-imaging due to their high UC efficiencies in spite of their toxic nature^1,50–54^.

### Cytotoxicity

**Figure 14:**
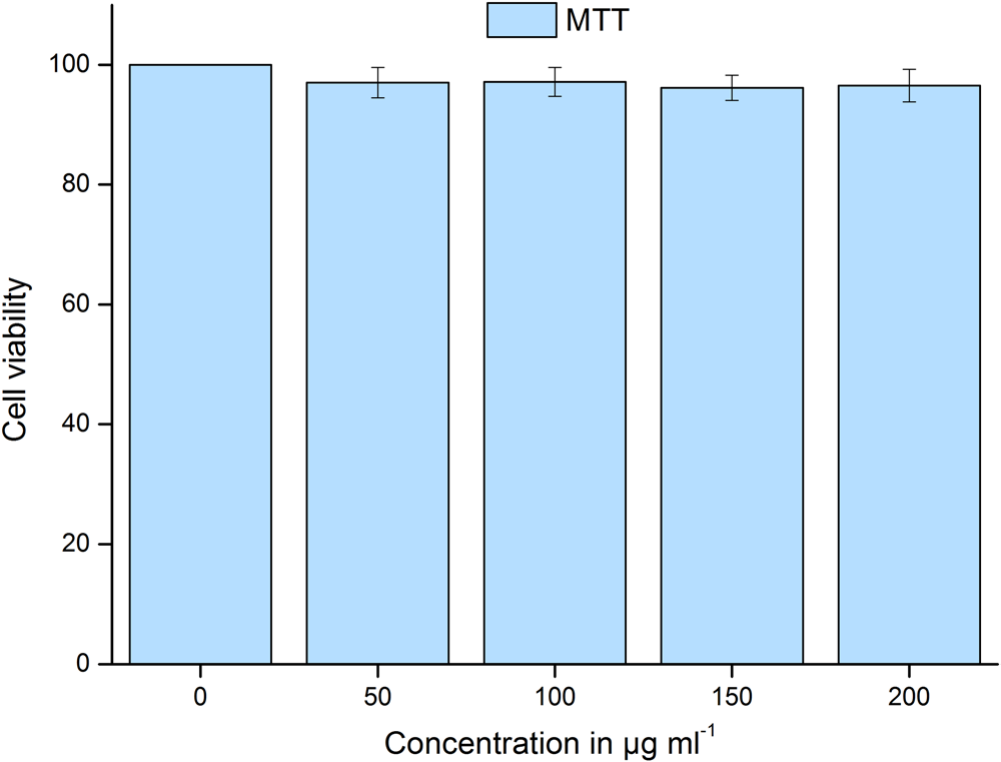
MTT ([3-(4,5-dimethylthiazol-2-yl)-2,5-diphenyltetrazoliumbromide]) assay in bluegill sunfish cells (BF-2) exposed to SLA powder at 0, 50, 100, 150 and 200 µg ml^−1^for 48 h. Data presented are mean ± SE (n = 15) of five independent experiments of three replicates each. Statistically no significant difference was observed as compared to controls (p ≤ 0.05).

To evaluate the cytotoxicity induced by SLA powders on aquatic biota, in *vitro* studies were conducted on BF-2 cell line. There was no significant cytotoxicity induced by SLA in BF-2 cells as evaluated using MTT assay. It is evident from the Fig. 14 that the SLA particles did not induce any significant loss of viability in BF-2 cell when compared to their respective control. This evaluation allow to conclude that the SLA material is not cytotoxic even at such a high concentration of 200 µg ml^−1^. Moreover, most of the researches have used BF-2 and other fish cells for toxicological evaluations of a number of contaminants. However, TiO_2_ and Ag particles were found to non-cytotoxic on BF-2 cells when exposed to simulated solar light and dark conditions^28^.

**Figure 15:**
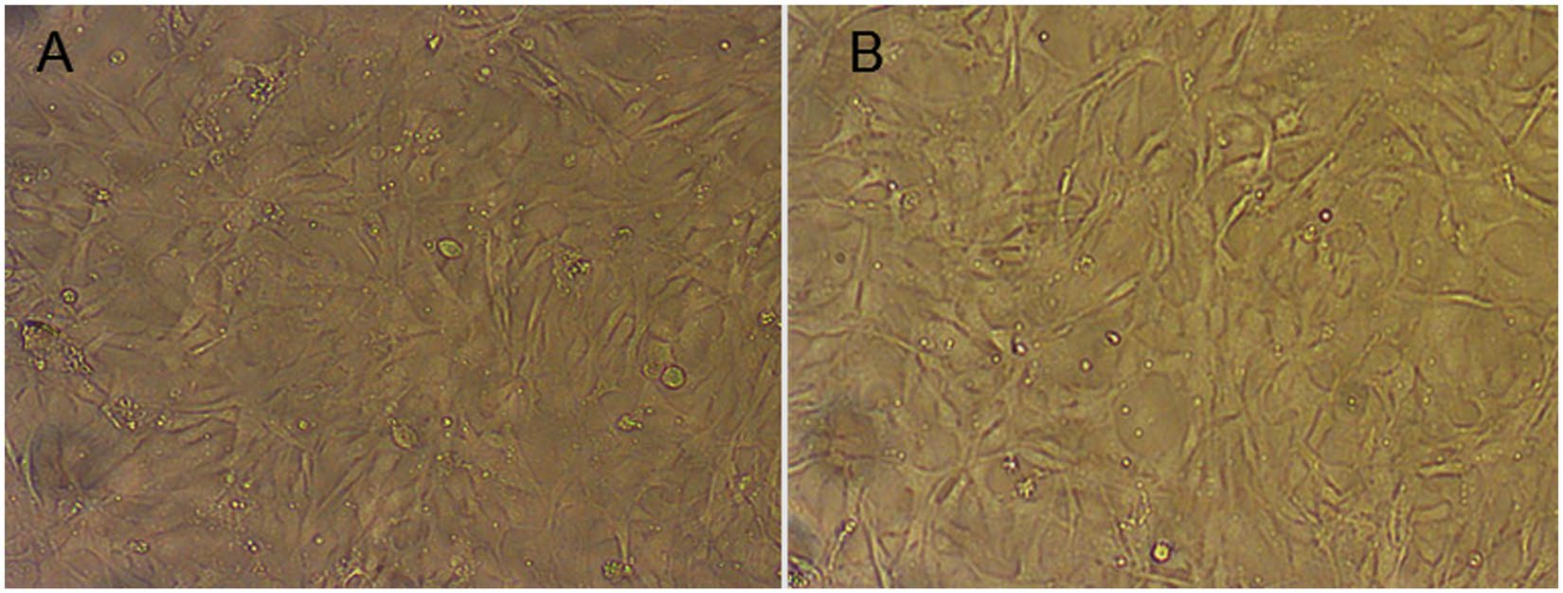
Bluegill sunfish cells (BF-2) (**A**) control (**B**) SLA powders treated at 200 µg ml^-1^ concentration for 48 h.

The BF-2 cells were exposed to 0 and 200 µg ml^−1^ of SLA particles for a time interval of 48 h and later observed under optical microscope. The SLA particles even at a concentration of 200 µg ml^−1^ did not induce any morphological changes in the exposed BF-2 cells until 48 h (Fig. 15). These results were in accordance to the MTT assays results which did not show any significant loss of viability when exposed to 200 µg ml^−1^ of SLA particles.

## Conclusions

In conclusion, strontium lanthanum aluminate (SrLaAl_3_O_7_) phosphors doped with Er^3+^ and Er^3+^/Yb^3+^ were prepared by one step Pechini method. The structural and morphological properties of the products were analyzed by X-ray diffraction, Raman spectroscopy and Scanning electron microscopy show that the materials prepared are structurally pure. NIR emission has been recorded with 980 nm and 514.5 nm laser radiation and show similar peak structure. The upconversion in SLA:Er and SLA:Er/Yb phosphors was found to be due to two-photon using power dependent upconversion spectra. Saturation of upconversion luminescence has been noticed at high powers in SLA:Er/Yb0.1 phosphor. Temperature dependent upconversion for SLA:Er and SLA:Er/Yb0.0.02 phosphors show exponential increase for G1 band whereas steady increase till 90 K and then decrease for the bands at G2 and R were observed. In other SLA:Er/Yb phosphors, G1 band increased exponentially whereas G2 and R quenched exponentially as usual. The maximum absolute sensitivity of SLA:Er was at 440 K and between 280–320 K for various SLA:Er/Yb variable concentrations. Among SLA:Er/Yb phosphors, the phosphor with highest intensity of UC emission exhibited higher relative sensitivity. Overall, SLA:Er/Yb0.1 phosphor exhibited better sensing qualities either in terms of ability to upconvert the radiation or sensing the temperature. The cytotoxicity evaluated for SLA particles on BF-2 fish cell found to be non-cytotoxic even at a concentration of 200 µg ml^−1^ and did not induce any morphological changes in the exposed cells until 48 h.

## Experimental

### Sample preparation

The SLA, SLA:Er and SLA:Er/Yb phosphors were synthesized by Pechini route. In a typical procedure, stoichiometric amounts of Strontium (Sr(NO_3_)_3_), Lanthanum (La(NO_3_)_3_), Aluminium (Al(NO_3_)_3_), Erbium (Er(NO_3_)_3_) and Ytterbium (Yb(NO_3_)_3_) nitrates were used as starting materials, citric acid as complexant agent and ethylene glycol used as chelating agent. The dopant ions, Er^3+^ and Yb^3+^, were expected to replace the La^3+^ ions with identical valence and similar ionic radius SLA matrix. The doping was done by fixing Er^3+^ concentration and varying Yb^3+^ concentration viz. SrLa_1−(x+y)_Al_3_O_7_:Er_x_Yb_y_ (x = 0.01, y = 0, 0.02, 0.04, 0.1 and 0.2) referred as SLA:Er, SLA:Er/Yb0.02, SLA:Er/Yb0.04, SLA:Er/Yb0.1 and SLA:Er/Yb0.2, respectively. The molar ratio of metal ions to citric acid was maintained as 1:1. A homogeneous solution was obtained by dissolving starting nitrates into distilled water, and the pH of the solution was adjusted to 1.5, 3 or 6.5 using ammonium hydroxide. The solution was dried at 100 °C for 12 h under continuous stirring. After complete evaporation of water, the gel was dried in oven at 250 °C for 3 h followed by heat treatment at 800 °C for 6 h to promote crystallinity. Finally, the collected powders were re-milled thoroughly by mixing acetone and pressed into pellets using a uniaxial pressure system.

### Measurements and characterization

The crystal structure of the prepared samples was identified by X-ray diffraction (XRD) using Philips PANalytical Xpert Pro analyser with Cu-Kα radiation. Raman spectra were measured by Jobin Yvon HR800 spectrometer with a 442 nm laser excitation source in reflection mode. The morphology of prepared phosphor powders was investigated by scanning electron microscopy (SEM) using TESCAN VEGA3 instrument.

Steady state UC spectra were recorded by a Spex 1704 monochromator equipped with a cooled Hamamatsu R928 photomultiplier. The samples were mounted in the cold finger of an optical cryostat, and the temperature was maintained in the range 14 to 300 K. A power-controllable 980 nm laser diode was used to exciting the samples. NIR PL was measured in FTIR Bruker IFS66V instrument with Ar^+^ ion laser (514.5 nm) as well as 980 nm laser diode.

BF-2 cells were obtained from American Type Culture Collection (ATCC # CCL91). The *in vitro* cytotoxicity has been performed on BF-2 fish cell obtained from the caudal fin of *L*. *macrochirus*. The cell line was maintained in Eagles minimum essential medium (EMEM) with non-essential amino acids supplemented with 10% fetal bovine serum, 100 U ml^−1^ pencillin, 100 µg ml^−1^ streptomycin, 1.25 µg ml^−1^ fungizone and 2 mM glutamine. Cultures were grown in 75 cm^2^ culture flask (Corning). The cultures were maintained at 23 ± 2 °C in an incubator supplied with 5% CO_2_. After the cell reaching 90% confluence, cells were harvested using 0.25% trypsin-EDTA and were sub-cultured in 96 well plates (5 × 10^3^ cells well^−1^) and were cultured over night at 23 °C under 5% CO_2_. Cells were allowed to attach to the surface for 24 h prior to treatment. After 24 h the cell culture medium was replaced with SLA particles at a concentration range of (50–200 µg ml^−1^) for 48 h. After 48 h the 96 well plates were subjected to centrifugation (100 xg for 5 min) using the swing bucket rotor (KUBOTA 6930, Japan) such that all the cells get deposited in the bottom of the plate and the supernatant is free from cells. After 48 h the entire medium was replaced with new medium containing MTT (5 mg ml^−1^) and incubated for 4 h at 21 °C in dark until a purple colour formazan product is formed. This product was dissolved in isopropanol and the absorbance was measured at 570 nm using a UV-Vis spectrophotometer (Spectra MAX Plus; Molecular Devices; supported by SOFT max PRO-3.0). Effects of SLA particles on cell viability were calculated using control cells (without particles).

Cells were plated in six well culture plates at a density of 2 × 10^4^ cells. After overnight incubation, the old medium is replaced with fresh medium containing 200 µg ml^−1^ of SLA particles for 48 h and the cells without any treatment served as control cells. After 48 h the cells were recovered and washed thoroughly with phosphate buffer saline and the morphological changes were observed using Polyvar, Reichert-Jung light microscope attached to a CCD camera (Sony CCDIRIS, Model No: SSC-M370CE) at 10x magnification.
